# Association of Endogenous Erythropoietin Levels and Iron Status With Cognitive Functioning in the General Population

**DOI:** 10.3389/fnagi.2022.862856

**Published:** 2022-04-08

**Authors:** Gizem Ayerdem, Matthijs J. Bosma, Joanna Sophia J. Vinke, Aaltje L. Ziengs, Adriaan R. E. Potgieser, Ron T. Gansevoort, Stephan J. L. Bakker, Martin H. De Borst, Michele F. Eisenga

**Affiliations:** ^1^Division of Nephrology, Department of Internal Medicine, University Medical Center Groningen, University of Groningen, Groningen, Netherlands; ^2^Department of Neuropsychology, University Medical Center Groningen, University of Groningen, Groningen, Netherlands; ^3^Department of Neurosurgery, University Medical Center Groningen, University of Groningen, Groningen, Netherlands

**Keywords:** erythropoietin (EPO), iron, cognitive functioning, general population, visual association test, ruff figural fluency test

## Abstract

**Background:**

Emerging data suggest that erythropoietin (EPO) promotes neural plasticity and that iron homeostasis is needed to maintain normal physiological brain function. Cognitive functioning could therefore be influenced by endogenous EPO levels and disturbances in iron status.

**Objective:**

To determine whether endogenous EPO levels and disturbances in iron status are associated with alterations in cognitive functioning in the general population.

**Materials and Methods:**

Community-dwelling individuals from the Prevention of Renal and Vascular End-Stage Disease (PREVEND) study, a general population-based cohort in Groningen, Netherlands, were surveyed between 2003 and 2006. Additionally, endogenous EPO levels and iron status, consisting of serum iron, transferrin, ferritin, and transferrin saturation were analyzed. Cognitive function was assessed by scores on the Ruff Figural Fluency Test (RFFT), as a reflection of executive function, and the Visual Association Test (VAT), as a reflection of associative memory.

**Results:**

Among 851 participants (57% males; mean age 60 ± 13 years), higher endogenous EPO levels were independently associated with an improved cognitive function, reflected by RFFT scores (ß = 0.09, *P* = 0.008). In multivariable backward linear regression analysis, EPO levels were among the most important modifiable determinants of RFFT scores (ß = 0.09, *P* = 0.002), but not of VAT scores. Of the iron status parameters, only serum ferritin levels were inversely associated with cognitive function, reflected by VAT scores, in multivariable logistic regression analysis (odds ratio, 0.77; 95% confidence interval 0.63–0.95; *P* = 0.02 for high performance on VAT, i.e., ≥11 points). No association between iron status parameters and RFFT scores was identified.

**Conclusion:**

The findings suggest that endogenous EPO levels and serum ferritin levels are associated with specific cognitive functioning tests in the general population. Higher EPO levels are associated with better RFFT scores, implying better executive function. Serum ferritin levels, but not other iron status parameters, were inversely associated with high performance on the VAT score, implying a reduced ability to create new memories and recall recent past. Further research is warranted to unravel underlying mechanisms and possible benefits of therapeutic interventions.

## Introduction

Erythropoietin (EPO) and iron are the primary regulators of red blood cell production. Besides being the fuel for erythropoiesis, EPO, and iron are known to express a myriad of non-hematopoietic effects (Nekoui and Blaise, 2017). An important non-hematopoietic effect concerns the maintenance of a normal physiological brain function (Ehrenreich et al., 2004; Ji et al., 2017). As a consequence, disturbances in EPO levels and iron status might negatively affect cognitive functioning, which is pivotal to focus, process information, and adapt or maintain a healthy lifestyle (Gill et al., 2020).

EPO receptors (EPOR) are prominently expressed in the brain in glial cells, neurons, and endothelial cells (Konishi et al., 1993; Digicaylioglu et al., 1995; Marti et al., 1996). EPO can pass the blood-brain barrier to exert its effect on the EPOR in the brain (Brines et al., 2000; Ehrenreich et al., 2004; Xenocostas et al., 2005). Moreover, in experimental models, astrocytes have been shown to produce and secrete EPO (Masuda et al., 1994). EPO promotes neural plasticity and has anti-inflammatory, anti-apoptotic, anti-oxidative, angiogenic, and stemcell-modulatory effects (Sirén et al., 2001; Gorio et al., 2002; Springborg et al., 2002; Buemi et al., 2003; Villa et al., 2003; Lykissas et al., 2007; Sargin et al., 2010; Girolamo et al., 2014; Nekoui et al., 2015). Therefore, EPO appears to have neuroprotective and neurotrophic properties, which in turn might hypothetically affect cognitive functioning (Wakhloo et al., 2020). Various studies focusing on different cerebral disease models support such a hypothesis, with some authors reporting that administration of recombinant human EPO (rhEPO) has a positive effect on cognitive functioning (Ehrenreich et al., 2008; Miskowiak et al., 2008, 2012, 2016; Sargin et al., 2010; Nekoui and Blaise, 2017).

Similarly, several studies have shown a relationship between serum iron levels and cognitive functioning (Miskowiak et al., 2012; Ji et al., 2017). Iron is a crucial part of many proteins including heme, iron sulfur clusters, and other functional groups (Schiepers et al., 2010). These proteins are essential for the formation of myelin surrounding axons and adenosine triphosphate in mitochondria, cell signaling, host defense, and nucleic acid replication and repair (Todorich et al., 2009; Mills et al., 2010; Evstatiev and Gasche, 2012). Iron is also crucially involved in the synthesis of several neurotransmitters, including tyrosine hydroxylase (dopamine and norepinephrine) and tryptophan (serotonin) (Moos et al., 2007; Todorich et al., 2009). As iron homeostasis is normally tightly regulated, iron deficiency, and overload affect enzymatic and structural proteins. Indeed, both iron deficiency and iron overload have been implicated in impaired cognitive functioning (Casanova and Araque, 2003; Miskowiak et al., 2012; Ji et al., 2017).

To date, the relationship between EPO levels and iron status with cognitive functioning has only been assessed in experimental models and relatively small sample size populations, often involving specific patient populations, e.g., having mood disorders (Beard et al., 1997; Milward et al., 1999; Weiskopf et al., 2006) questioning the role of endogenous EPO levels and iron status with cognitive functioning in the general population. Hence, we aimed to assess in a large population-based cohort the association between endogenous EPO levels and iron status parameters with cognitive functioning as reflected by two cognitive tests, i.e., the Ruff Figural Fluency Test (RFFT) and the Visual Association Test (VAT).

## Materials and Methods

### Study Design and Population

**FIGURE 1:**
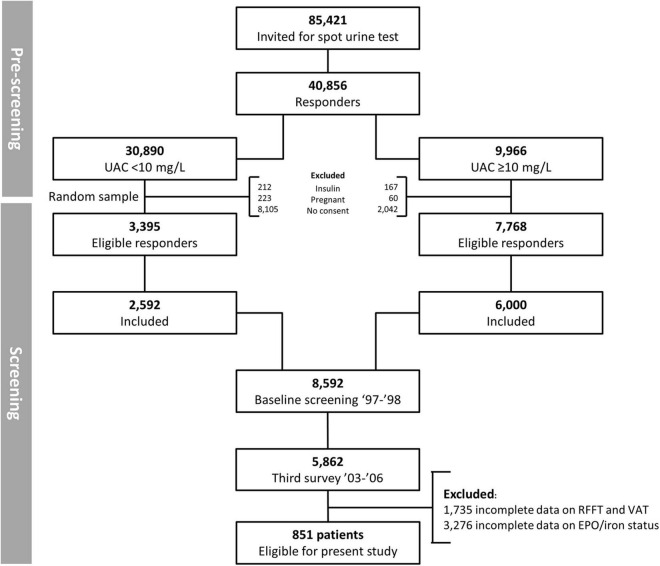
Flowchart of 851 PREVEND participants selected for final analysis.

For this study, we used the PREVEND (Prevention of REnal and Vascular ENd stage Disease) database, a general population-based cohort study. In 1997 and 1998, all inhabitants from the city of Groningen, between the age of 28–75 years (*n* = 85,421), were sent a short questionnaire and a vial to collect a first-morning void urine sample. 40,856 (48%) people responded. Individuals with insulin-dependent Diabetes Mellitus and pregnant women were excluded. Six thousand subjects with a urinary albumin excretion ≥ 10 mg/L and 2,592 randomly selected subjects (control group) with a urinary albumin excretion < 10 mg/L completed the screening protocol and formed the PREVEND cohort baseline (*n* = 8,592). We used the third survey of PREVEND, which took place between 2003 and 2006. Of these participants, multiple blood samples were collected in which, among others, EPO levels and iron status parameters were measured. Two tests reflecting certain cognitive domains (i.e., RFFT and VAT) were introduced during the same survey. For current analysis, we included 851 patients with data available on EPO levels, iron status, and both cognitive tests (as depicted in Figure 1). The PREVEND study protocol was approved by the institutional medical ethical review board of the University Medical Center Groningen and was conducted in accordance with the Helsinki declaration. All subjects provided written informed consent.

### Data Collection and Definitions

The procedures at each examination in the PREVEND study have been described in detail previously (Hillege et al., 2001). In short, upon entry into the study, all participants completed a questionnaire regarding demographics, current diagnoses, medical history, smoking habits, alcohol consumption, and medication use. Information on medication use was combined with information from a pharmacy-dispensing registry. Educational level was defined as low (primary education or intermediate vocational education), middle (higher secondary education), and high (higher vocational education and university). Antihypertensives included diuretics, β–blockers, calcium channel blockers, and renin-angiotensin system inhibitors.

### Cognitive Function Testing

#### Ruff Figural Fluency Test

The RFFT measures a subject’s ability to produce novel figures utilizing five different dot configurations (Ruff et al., 1987). Participants were instructed to produce as many unique designs as possible using at least two of the dots in a 5-dot matrix. The lowest score is 0 points, the highest and best score is 175 points. The RFFT is a sensitive test for executive cognitive abilities such as non-verbal fluency, planning strategies, task shifting, selective attention, response evaluation, and response suppression, which are necessary to coordinate this process (Mulder et al., 2006). It has been shown to be sensitive to early changes in cognitive function in young as well as middle-aged people (Foster et al., 2005). A reduced ability to produce novel figures can indicate a disability in executive function in general and has been linked to processes in the frontal lobe, most prominently in the right frontal lobe (Ruff et al., 1994; Mulder et al., 2006).

#### Visual Association Test

The VAT is a brief episodic memory test presenting six paired pictures of two interacting objects where one has to name the missing object on a cue card which has been shown before. One point is given if the missing object is correctly identified. The minimum score is 0 points; the maximum score is 12 points. The test is used to detect anterograde amnesia and related syndromes, usually associated with atrophy of the (medial-temporal areas of the) limbic system. It is hypothesized that a low score on the VAT is related to impaired ability in coding new information or, less likely, in short-term memory (Lindeboom et al., 2002).

### Measurements

Fresh fasting blood samples were collected in the morning from all participants and stored at −80°C. EPO was measured using an immunoassay based on chemiluminescence (Immulite EPO assay, DPC, Los Angeles, CA, United States). Serum iron, ferritin, and transferrin were measured using colorimetrix assay, immunoassay, or immunoturbidimetric assay (Roche Diagnostics, Mannheim, Germany), respectively. Transferrin saturation (TSAT,%) was calculated as 100 × serum iron (μmol/L)÷(25 × transferrin[g/L]). A Coulter Counter STKS sum was used to measure hemoglobin (g/dL) (Coulter Corporation, Miami, FL, United States). Serum creatinine was measured using an enzymatic method on a Roche Modular analyzer (Roche Diagnostics). The Chronic Kidney Disease Epidemiology Collaboration (CKD-EPI) formula was used to calculate the estimated glomerular filtration rate (eGFR) (Levey et al., 2009). Urinary albumin concentration was determined by nephelometry (BN II, Dade Behring Diagnostica, Marburg, Germany). Urinary albumin excretion (UAE) was calculated as the average UAE in the two consecutive 24-h urine collections. Body mass index (BMI) was calculated as the ratio of weight divided by height squared (kg/m^2^). High-sensitivity C-reactive protein (hs-CRP), cholesterol, high- density lipoprotein (HDL), and low-density lipoprotein (LDL) were measured using routine laboratory procedures.

### Statistical Analysis

Data were analyzed using IBM SPSS, version 23.0 (SPSS, Chicago, IL, United States) and R version 4.0.2 (Vienna, Austria). Baseline characteristics were described as means with standard deviation when normally distributed and medians with interquartile range when distributions were skewed. Categorical variables were reported using numbers (percentage). We described baseline characteristics both for the total population and across quartiles of EPO and ferritin levels. Differences between the quartiles were calculated using analysis of variance (ANOVA), Kruskal-Wallis test or χ2-test, as appropriate. In regression analysis, we determined if serum EPO levels and iron status parameters can be regarded as important determinants of cognitive capacity domains measured by the RFFT and VAT. Univariable linear regression analysis was performed of all included factors with RFFT as the dependent variable. Subsequently, we performed multivariable-adjusted models and multivariable backward linear regression analysis (entry and exit level set at *P* < 0.2 and *P* < 0.1, respectively). In all regression analyses, skewed variables were naturally log-transformed. In the multivariable model, we adjusted for age, sex, education, BMI, eGFR, and urinary albumin excretion (model 1); for systolic blood pressure, alcohol use, smoking, hemoglobin, hs-CRP, serum HDL, and serum LDL levels (model 2); and for history of a myocardial or cerebrovascular event, diabetes mellitus, and the use of antihypertensives, and lipid lowering drugs (model 3). Due to the skewed distribution, the VAT scores were dichotomized and divided at the median into high performance (≥11 points) and low performance (≤10 points), in line with previous investigations of the VAT score (Joosten et al., 2014). The association of EPO levels and iron status parameters were evaluated by logistic regression analysis in a similar way. In multiple regression analyses, iron status parameters were assessed separately due to multi-collinearity between the iron status parameters. The contribution of EPO levels was reported with ferritin levels as only iron status parameter in all models. Statistical significance was considered as a two-tailed *p*-value of <0.05.

## Results

### Study Population

We included 851 participants (57% males) with a mean age of 60.3 ± 13.0 years. Participants had a mean BMI of 27.1 ± 3.9 kg/m^2^ and an eGFR of 85.5 ± 19.5 ml/min/1.73m^2^. A majority of the participants [412 (48%)] registered a low educational level, whereas 226 (27%) and 213 (25%) had a middle and high educational level, respectively. The median EPO level in the total population was 7.8 (5.9–10.1) IU/L; median ferritin concentration was 117 (58–197) μg/L; mean iron level was 16.3 ± 5.2 μmol/L; mean transferrin level was 2.5 ± 0.4 g/L; and mean TSAT was 26.4 ± 9.2%. Further baseline demographics, clinical characteristics, and laboratory parameters according to quartiles of EPO and ferritin levels in the total population are depicted in Tables 1, 2, respectively.

**TABLE 1:** Baseline characteristics of 851 community-dwelling subjects according to quartiles of EPO levels.

		EPO quartiles				
	Overall	Q1	Q2	Q3	Q4	*p*-value
	*N* = 851	213	213	214	211	
	(range)	(1.6–5.9)	(5.9–7.8)	(7.8–10.1)	(10.2–99.4)	
Cognitive tests						
VAT	10 (8–11)	10 (8–11)	10 (8–11)	10 (8–11)	10 (8–11)	0.79
High performance on VAT (n,%)	297 (35%)	82 (38%)	69 (32%)	75 (35%)	71 (34%)	0.58
RFFT	62 ± 25	63.9 ± 26.2	61.7 ± 24.3	62.1 ± 25.5	61.9 ± 25.7	<0.001
Age	60.3 ± 13	57.5 ± 12.6	59.7 ± 12.9	60.7 ± 12.8	63.2 ± 13.1	<0.001
Male sex (n,%)	485 (57%)	112 (53%)	118 (55%)	132 (62%)	123 (58%)	0.26
Education						0.71
Low (n,%)	412 (48%)	103 (48%)	104 (49%)	105 (49%)	100 (47%)	
Middle (n,%)	226 (27%)	50 (23%)	54 (25%)	63 (29%)	59 (28%)	
High (n,%)	213 (25%)	60 (28%)	55 (26%)	46 (21%)	52 (25%)	
Medication use						
Antihypertensives (n,%)	254 (31%)	47 (22%)	57 (28%)	67 (32%)	83 (40%)	0.001
Lipid lowering (n,%)	141 (17%)	19 (9%)	37 (18%)	37 (18%)	48 (23%)	0.002
Iron suppletion (n,%)	2 (<1%)	0 (0%)	2 (1%)	0 (0%)	0 (0%)	0.11
Health behavior and medical history						
BMI (kg/m^2^)	27.2 ± 3.7	26.3 ± 3.5	26.9 ± 3.8	27.1 ± 4.1	28 ± 4.1	<0.001
Systolic blood pressure (mmHg)	130 ± 19	125 ± 17	129 ± 18	130 ± 17	135 ± 21	<0.001
Alcohol use (n,%)	641 (76%)	175 (83%)	170 (81%)	152 (72%)	144 (68%)	0.001
Smoking (n,%)	164 (19%)	55 (26%)	39 (18%)	36 (17%)	34 (16%)	0.04
Cardiac event*^a^* (n.%)	24 (3%)	4 (2%)	5 (2%)	6 (3%)	9 (4%)	0.48
Cerebrovascular event*^b^* (n,%)	15 (2%)	2 (1%)	7 (3%)	3 (1%)	3 (1%)	0.26
Diabetes Mellitus*^c^* (n,%)	82 (10%)	18 (9%)	19 (9%)	16 (8%)	29 (14%)	0.13
Laboratory measurements						
EPO (IU/L)	7.8 (5.9–10.1)	4.8 (4.1–5.4)	6.9 (6.4–7.3)	8.9 (8.3–9.3)	12.9 (11.4–15.7)	–
Iron status						
Ferritin (μg/L)	117 (58–197)	125 (77–213)	117 (70–197)	119.5 (58–204)	93 (32–178)	0.001
Iron (μmol/L)	16.3 ± 5.2	17.4 ± 4.9	16.9 ± 5.2	16.2 ± 4.8	14.7 ± 5.5	<0.001
Transferrin (g/L)	2.5 ± 0.4	2.5 ± 0.3	2.5 ± 0.4	2.5 ± 0.4	2.6 ± 0.5	0.05
Transferrin saturation (%)	26 ± 9	28 ± 9	28 ± 9	26 ± 8	23 ± 10	<0.001
Hemoglobin (g/dL)	13.9 ± 1.1	14.0 ± 1.1	14.0 ± 1.1	13.9 ± 1.1	13.4 ± 1.3	<0.001
MCV (fL)	90.1 ± 4.2	89.4 ± 3.8	90.2 ± 3.6	90.1 ± 4	90.7 ± 5.2	0.02
eGFR (ml/min/1.73m^2^)	86 ± 20	89 ± 19	87 ± 19	86 ± 19	81 ± 21	<0.001
Urinary albumin excretion (mg/24 h)	9.5 (6.6–20.8)	9.1 (6.6–15.2)	9.4 (6.5–17.8)	9.2 (6.4–23.3)	11 (7.1–28.3)	0.008
hs-CRP (mg/L)	1.3 (0.6–2.4)	1.2 (0.6–2.1)	1.2 (0.6–2.2)	1.2 (0.6–2.1)	1.5 (0.7–3.7)	0.001
Cholesterol (mmol/L)	5.4 ± 1.1	5.6 ± 1	5.4 ± 1.1	5.4 ± 1	5.2 ± 1.1	0.003
HDL (mmol/L)	1.4 ± 0.4	1.4 ± 0.4	1.4 ± 0.4	1.4 ± 0.4	1.4 ± 0.4	0.70
LDL (mmol/L)	1.1 ± 0.4	1.1 ± 0.4	1.1 ± 0.4	1 ± 0.3	1 ± 0.4	0.04
Triglycerides (mmol/L)	1.2 (0.9–1.7)	1.3 (1–1.8)	1.2 (0.9–1.7)	1.3 (0.9–1.6)	1.2 (0.9–1.7)	0.19

*Data are expressed as mean ± standard deviation, median (interquartile range), or proportion n (%). Abbreviations: BMI, body mass index; eGFR, estimated glomerular filtration rate; EPO, erythropoietin; hs-CRP, high-sensitivity C-reactive protein; HDL, high-density lipoproteins; LDL, low-density lipoproteins; MCV, mean corpuscular volume; RFFT, Ruff Figural Fluency Test; VAT, Visual Association Test. ^a^Cardiac event included a history of myocardial infarction and ischemic heart disease, ^b^cerebrovascular event included a history of subarachnoid hemorrhage, intra-cerebral hemorrhage, other and unspecified intracranial hemorrhage, and occlusion and stenosis of pre-cerebral or cerebral arteries, ^c^not-insulin dependent diabetes mellitus.*

**TABLE 2:** Baseline characteristics of 851 community-dwelling subjects according to quartiles of ferritin levels.

		Ferritin quartiles				
	Overall	Q1	Q2	Q3	Q4	*P*-value
	*N* = 851	214	214	213	210	
	(range)	(4.0–58.0)	(59.0–117.0)	(118.0–197.0)	(198.0–1309.0)	
Cognitive tests						
VAT	10 (8–11)	10 (9.0–11)	10 (8–11)	10 (8–11)	9 (7–10)	<0.001
High performance on VAT (n,%)	297 (35%)	99 (46%)	74 (35%)	72 (34%)	52 (25%)	<0.001
RFFT	62 ± 25	67.5 ± 26.2	66.4 ± 25.2	58.4 ± 25.4	57.3 ± 23.2	<0.001
Age	60.3 ± 13	55.4 ± 13.3	59.4 ± 12.4	63.2 ± 12.3	63.2 ± 12.4	<0.001
Male sex (n,%)	485 (57%)	75 (35%)	118 (55%)	131 (62%)	131 (62%)	<0.001
Education						0.21
Low (n,%)	412 (48%)	94 (44%)	103 (48%)	111 (52%)	104 (50%)	
Middle (n,%)	226 (27%)	58 (27%)	54 (25%)	63 (30%)	51 (24%)	
High (n,%)	213 (25%)	62 (29%)	57 (27%)	39 (18%)	55 (26%)	
Medication use						
Antihypertensives (n,%)	254 (31%)	39 (19%)	51 (25%)	76 (36%)	88 (43%)	<0.001
Lipid lowering (n,%)	141 (17%)	15 (7%)	35 (17%)	43 (20%)	48 (24%)	<0.001
Iron suppletion (n,%)	2 (<1%)	1 (<1%)	0 (0%)	1 (<1%)	0 (0%)	0.57
Health behavior and medical history						
BMI (kg/m^2^)	27.1 ± 3.9	26.2 ± 4	26.6 ± 3.7	27.2 ± 3.7	28.5 ± 3.8	<0.001
Systolic blood pressure (mmHg)	130 ± 19	125 ± 18	127 ± 17	132 ± 20	135 ± 18	<0.001
Alcohol use (n,%)	641 (76%)	143 (67%)	170 (80%)	160 (76%)	168 (81%)	0.002
Smoking (n,%)	164 (19%)	50 (23%)	54 (25%)	35 (17%)	25 (12%)	0.001
Cardiac event*^a^* (n.%)	24 (3%)	5 (2%)	8 (4%)	4 (2%)	7 (3%)	0.63
Cerebrovascular event*^b^* (n,%)	15 (2%)	2 (1%)	3 (1%)	5 (2%)	5 (2%)	0.60
Diabetes Mellitus*^c^* (n,%)	82 (10%)	15 (7%)	12 (6%)	20 (10%)	35 (17%)	<0.001
Laboratory measurements						
EPO (IU/L)	7.8 (5.9–10.1)	8.6 (6.4–12.3)	7.4 (5.8–9.6)	7.5 (5.7–9.7)	7.7 (5.7–9.7)	<0.001
Iron status						
Ferritin (μg/L)	117 (58–197)	32.5 (22–45)	88 (75–101)	150 (131–173)	273 (230–355)	–
Iron (μmol/L)	16.3 ± 5.2	14.9 ± 5.9	16.1 ± 4.5	16.6 ± 4.9	17.8 ± 5.1	<0.001
Transferrin (g/L)	2.5 ± 0.4	2.8 ± 0.4	2.5 ± 0.3	2.4 ± 0.3	2.4 ± 0.3	<0.001
Transferrin saturation (%)	26 ± 9	22 ± 9	26 ± 8	28 ± 9	30 ± 10	<0.001
Hemoglobin (g/dL)	13.9 ± 1.1	13.2 ± 1.1	13.9 ± 1.1	13.9 ± 1.1	14.2 ± 1.1	<0.001
MCV (fL)	90.1 ± 4.2	89.3 ± 4.4	90.4 ± 4.0	90.0 ± 4.1	90.8 ± 4.2	0.003
eGFR (ml/min/1.73m^2^)	86 ± 20	90 ± 19	86 ± 20	82 ± 19	83 ± 20	<0.001
Urinary albumin excretion (mg/24 h)	9.5 (6.6–20.8)	8.8 (6.2–17.6)	8.7 (6.6–15.2)	9.5 (6.5–20.6)	12.2 (7.4–28.3)	<0.001
hs-CRP (mg/L)	1.3 (0.6–2.4)	1.2 (0.6–2.6)	1.2 (0.6–2.3)	1.25 (0.61–2.12)	1.4 (0.7–3.2)	0.17
Cholesterol (mmol/L)	5.4 ± 1.1	5.4 ± 1.2	5.5 ± 1.1	5.4 ± 1.0	5.4 ± 1.0	0.68
HDL (mmol/L)	1.4 ± 0.4	1.5 ± 0.4	1.5 ± 0.4	1.3 ± 0.3	1.3 ± 0.3	<0.001
LDL (mmol/L)	1.1 ± 0.4	1.0 ± 0.4	1.1 ± 0.4	1.1 ± 0.4	1.1 ± 0.4	<0.001
Triglycerides (mmol/L)	1.2 (0.9–1.7)	1.1 (0.8–1.5)	1.2 (0.9–1.5)	1.3 (1.0–1.7)	1.4 (1.1–1.9)	<0.001

*Data are expressed as mean ± standard deviation, median (interquartile range) or proportion n (%). Abbreviations: BMI, body mass index; eGFR, estimated glomerular filtration rate; EPO, erythropoietin; hs-CRP, high-sensitivity C-reactive protein; HDL, high-density lipoproteins; LDL, low-density lipoproteins; MCV, mean corpuscular volume; RFFT, Ruff Figural Fluency Test; VAT, Visual Association Test. ^a^Cardiac event included a history of myocardial infarction and ischemic heart disease, ^b^cerebrovascular event included a history of subarachnoid hemorrhage, intra-cerebral hemorrhage, other and unspecified intracranial hemorrhage, and occlusion and stenosis of pre-cerebral or cerebral arteries, ^c^not-insulin dependent diabetes mellitus.*

### Ruff Figural Fluency Test and Visual Association Test Scores in the Total Population

Participant scored an average of 62 ± 25 points on the RFFT. If compared to the norm score, 672 (79%) scored average on the RFFT, 85 (10%) above average, 68 (8%) below average, and 26 (31%) participants had a deviant. Participants obtained a median VAT score of 10 points (IQR = 8–11). Considering the norm, 684 (80%) participants scored average on the VAT, 107 (12.6%) above average, 42 (5%) below average, and 18 (2%) people had a deviant.

### Erythropoietin, Iron Status, and Ruff Figural Fluency Test Score

Across quartiles of serum EPO levels, we noticed a significant inverse association with RFFT score. Individuals in the lowest quartile of EPO levels had 63.9 ± 26.2 points, whereas participants in the upper quartile of EPO scored 61.9 ± 25.7 points on the RFFT (*P* < 0.001). Similarly, we identified a significant inverse association, even more pronounced, across quartiles of ferritin levels. Participants within the lowest quartile of ferritin obtained 67.5 ± 26.2 points, whereas participants in the highest quartile of ferritin obtained 57.3 ± 23.2 points on the RFFT (*P* < 0.001).

In multivariable linear regression analysis, EPO levels were significantly associated with RFFT scores independent of potential confounders (fully adjusted ß = 0.09, *P* = 0.008 as depicted in model 3; Table 3). None of the iron status parameters, including serum ferritin, were significantly associated with RFFT scores.

**TABLE 3:** Univariate and backward linear regression analyses of potential determinants of RFFT scores.

	Univariate analysis		Backward analysis	
	Std. β	*P*-value	Std. β	*P*-value
Age	–0.54	<0.001	–0.44	<0.001
Male sex	0.02	0.54		
**Education**				
Low	–0.35	<0.001	***Ref.***	
Middle	0.08	0.02	0.14	<0.001
High	0.32	<0.001	0.26	<0.001
**Medication use**				
Antihypertensives	–0.30	<0.001	–0.05	0.13
Lipid lowering	–0.15	<0.001		
Iron suppletion	–0.24	0.49		
**Health behavior and medical history**				
BMI (kg/m^2^)	–0.24	<0.001	–0.05	0.09
Systolic blood pressure (mmHg)	–0.28	<0.001		
Alcohol use (n,%)	0.25	<0.001	0.11	<0.001
Smoking (n,%)	–0.03	0.42		
Cardiac event (n,%)	–0.11	0.001		
Cerebrovascular event (n,%)	–0.05	0.12		
Diabetes Mellitus (n,%)	–0.19	<0.001		
**Laboratory measurements**				
EPO (IU/L)^a^	–0.03	0.45	0.09	0.002
Iron status				
Ferritin (μg/L)	–0.18	<0.001		
Iron (μmol/L)	–0.01	0.67		
Transferrin (g/L)	0.07	0.05		
Transferrin saturation (%)	–0.04	0.30		
Hemoglobin (mmol/L)	–0.008	0.81		
MCV (fL)	–0.006	0.86		
eGFR (ml/min/1.73m^2^)	0.41	<0.001		
Urinary albumin excretion (mg/24 h)	–0.22	<0.001		
hs-CRP (mg/L)	–0.23	<0.001	–0.05	0.15
Cholesterol (mmol/L)	0.05	0.18	0.05	0.09
HDL (mmol/L)	0.15	<0.001	0.05	0.11
LDL (mmol/L)	–0.09	0.01		
Triglycerides (mmol/L)	–0.02	0.67		

*Abbreviations: BMI, body mass index; eGFR, estimated glomerular filtration rate; EPO, erythropoietin; hs-CRP, high-sensitivity C-reactive protein; HDL, high-density lipoproteins; LDL, low-density lipoproteins; MCV, mean corpuscular volume; Std. β, Standardized beta; RFFT, Ruff Figural Fluency Test; Ref., reference category.*

*^a^Adjusted for only ferritin as iron status parameter.*

In multivariable linear backward regression analysis, EPO levels were among the main determinants of RFFT (β = 0.09, *P* = 0.002). In contrast, none of the iron status parameters was significantly associated with RFFT (Table 4).

**TABLE 4:** Multivariate linear regression analyses of the association of individual iron status parameters and erythropoietin with RFFT score.

	Model 1^a^		Model 2^b^		Model 3^c^	
	Std. β	*P*-value	Std. β	*P*-value	Std. β	*P*-value
EPO (IU/L)^d^	0.07	0.03	0.09	0.005	0.09	0.008
Ferritin (ug/L)	0.004	0.90	–0.01	0.66	–0.02	0.48
Iron (umol/L)	–0.01	0.71	–0.02	0.50	–0.03	0.38
Transferrin (g/L)	–0.007	0.79	–0.004	0.88	0.005	0.87
TSAT (%)	–0.01	0.67	–0.02	0.49	–0.04	0.26

*Abbreviations: BMI, body mass index; CI, confidence interval; eGFR, estimated glomerular filtration rate; EPO, erythropoietin; hs-CRP, high-sensitivity C-reactive protein; HDL, high-density lipoproteins; LDL, low-density lipoproteins; RFFT, Ruff figural fluency test; Std. β, standardized beta; TSAT, transferrin saturation.*

*^a^Model 1: adjusted for age, sex, education, BMI, eGFR, and urinary albumin excretion.*

*^b^Model 2: Model 1 + adjustment for systolic blood pressure, alcohol use, smoking, hemoglobin, hs-CRP, serum HDL, serum LDL.*

*^c^Model 3: Model 2 + adjustment for history of cerebrovascular event, diabetes mellitus, and use of antihypertensives, and lipid-lowering drugs.*

*^d^Adjusted for only ferritin as iron status parameter.*

### Erythropoietin, Iron Status, and Visual Association Test

Across quartiles of EPO, we identified no significant differences in VAT score (*P* = 0.79). In contrast, we identified that the prevalence of high performance scores (i.e., ≥11 points) was significantly different across quartiles of ferritin levels. Of the participants within the lowest quartile of ferritin, 46% had a high performance score, whereas only 25% of the participants within the upper quartile of ferritin had a high performance score (*P* < 0.001).

In multivariable logistic regression analysis, EPO levels were not significantly associated with a high performance on the VAT. Of the iron status parameters, only serum ferritin was significantly inversely associated with a high performance on the VAT (fully adjusted OR, 0.77; 95% CI 0.63 – 0.95; *P* = 0.02 as depicted in model 3, Table 5). In contrast, serum iron, transferrin, and TSAT were not associated with a high performance on the VAT score (Table 5).

**TABLE 5:** Binomial logistic regression analyses of the association of individual iron status parameters and erythropoietin with a high performance on the VAT score.

	Model 1^a^			Model 2^b^			Model 3^c^		
	OR	95% CI	*P*-value	OR	95% CI	*P*-value	OR	95% CI	*P*-value
EPO (IU/L)^d^	0.92	0.65–1.31	0.65	1.05	0.71–1.54	0.81	1.01	0.68–1.51	0.95
Ferritin (ug/L)	0.79	0.65–0.95	0.01	0.78	0.63–0.95	0.01	0.77	0.63–0.95	0.02
Iron (umol/L)	0.99	0.96–1.02	0.37	0.99	0.96–1.02	0.37	0.98	0.95–1.02	0.27
Transferrin (g/L)	1.15	0.77–1.72	0.50	1.04	0.68–1.59	0.86	1.02	0.66–1.58	0.91
TSAT (%)	0.99	0.97–1.01	0.32	0.99	0.98–1.01	0.46	0.99	0.97–1.01	0.33

*Abbreviations: BMI, body mass index; CI, confidence interval; eGFR, estimated glomerular filtration rate; EPO, erythropoietin; hs-CRP, high-sensitivity C-reactive protein; HDL, high-density lipoproteins; LDL, low-density lipoproteins; OR, odds ratio; TSAT, transferrin saturation; VAT, Visual association test.*

*^a^Model 1: adjusted for age, sex, education, BMI, eGFR, and urinary albumin excretion.*

*^b^Model 2: Model 1 adjustment for systolic blood pressure, alcohol use, smoking, hemoglobin, hs-CRP, serum HDL, and serum LDL.*

*^c^Model 3: Model 2 adjustment for history of cerebrovascular event, diabetes mellitus, and use of antihypertensives, and lipid-lowering drugs.*

*^d^Adjusted for only ferritin as iron status parameter.*

## Discussion

In this study, we show that in the general population higher endogenous EPO levels are associated with better executive function, reflected by RFFT scores, whereas higher ferritin levels, but not other iron status parameters, are associated with a lower VAT score, reflecting associative memory. To the best of our knowledge, this is the first study to show associations between serum EPO and ferritin levels and specific domains of cognitive functioning in the general population.

It has been suggested that EPO exerts a protective effect on cognitive functioning due to its neuroprotective and neurotrophic potential (Sirén et al., 2001; Gorio et al., 2002; Springborg et al., 2002; Buemi et al., 2003; Villa et al., 2003; Lykissas et al., 2007; Sargin et al., 2010; Girolamo et al., 2014; Nekoui et al., 2015). The latter has mainly been concluded based on studies in which exogenous EPO was administered (Ehrenreich et al., 2008; Nekoui and Blaise, 2017). Specifically, these studies showed an increase in cognitive function test scores. Here, we demonstrate that higher endogenous EPO levels are associated with better RFFT scores, reflecting improved executive function with improved capabilities such as non-verbal fluency, planning strategies, task shifting, selective attention, response evaluation, and response suppression, which are necessary to coordinate this process (Mulder et al., 2006). This is in line with the hypothesis based on earlier findings of EPO and EPOR expression in (mammalian) brain areas related to executive functioning, and with studies by Ehrenreich et al. and Miskowiak et al. in which exogenous EPO increased several of these (or related) executive functions (Digicaylioglu et al., 1995; Marti et al., 1996; Ehrenreich et al., 2007a,b; Miskowiak et al., 2008, 2014; Nowrangi et al., 2014). Importantly, the effect of high endogenous EPO levels on RFFT scores seems to be independent of the effect of EPO on hematopoiesis, as adjustment for hemoglobin levels did not alter the association. The latter suggests a direct neurobiological effect of EPO on cognition, most likely because of its neuroprotective and neurotrophic potential as an underlying mechanism, which is in line with the long-term impact of EPO on cognition in several other studies (Ehrenreich et al., 2007a,b; Miskowiak et al., 2014).

The exact biological relevance of our endogenous serum EPO levels is difficult to interpret in the absence of a direct measurement of EPO in the brain. EPO is known to cross the blood-brain barrier by active translocation, most likely *via* EPOR expressed in the brain vasculature pattern (Brines et al., 2000). The studies who investigated the neuroprotective and neurotrophic potential of exogenous EPO administered high-dose EPO to induce significant elevations in cerebrospinal fluid and brain EPO levels to improve cognitive function. However, the importance of endogenous circulating EPO levels has also previously been shown in children with malaria where high EPO levels were associated with a reduced risk of neurological sequelae (Casals-Pascual et al., 2008). Similarly, in a recent study, Shim et al. (2021) showed the relationship between circulating EPO levels and attention deficit hyperactivity disorder (ADHD) rating scale in children with ADHD and healthy controls.

We did not find an association between endogenous EPO levels and performance on the VAT, suggesting that endogenous EPO levels did not affect the ability to create new memories and to recall the recent past. Aside from a few exceptions, this runs counter to reports that exogenous EPO improves certain memory-related abilities, as can be seen through upregulation of activity during memory tasks (Ehrenreich et al., 2007b; Miskowiak et al., 2008, 2009, 2014) and upregulation of memory-related brain areas during a memory task (Miskowiak et al., 2007, 2016). With evidence of EPO and EPOR being present in brain areas related to memory, e.g., the hippocampus and areas within the temporal lobe, we expected a positive association of endogenous EPO levels on the VAT score (Digicaylioglu et al., 1995; Marti et al., 1996; Rombouts et al., 1997). The discrepancy between our currently identified results and those from other studies might be related to the use of different populations in earlier studies, which focused on subjects with depression or schizophrenia.

Moreover, and more likely, the VAT is designed to detect anterograde amnesia and related syndromes. It is a relatively simple task with a small range in scores compared to the RFFT and less suitable to detect subtle differences in memory ability.

Regarding iron status, we did not identify a U-shaped association between iron status and cognitive function, as might have been expected, since previous studies related both a low and high serum iron to a decline in certain cognitive abilities (Lam et al., 2008; Schiepers et al., 2010; Ji et al., 2017). However, we did find that higher ferritin levels increased the risk of a low performance on the VAT. This suggests that increased ferritin levels in the general population are associated with a diminished ability to create new memories and recall the recent past. Since serum ferritin is not related to the iron content in brain regions involved in memory abilities, like the hippocampus and temporal cortex (Gao et al., 2017), the underlying mechanism is not clear. Our finding is contrary to the few previous studies on serum ferritin levels and cognition. Schiepers et al. (2010) found that higher serum ferritin was associated with decreased speed of cognitive functioning, but did not find serum ferritin to be related to memory processes. Milward et al. (2010) found that abnormal levels of ferritin were not associated with global cognitive performance or executive function. When considering serum ferritin as a proxy for body iron stores, our findings are in line with research by Lam et al. (2008), in which very high serum iron concentrations were associated with poorer outcomes on tests measuring short and long-term memory processes. However, caution is warranted to consider serum ferritin solely as surrogate for body iron stores. Serum ferritin is also an acute-phase reactant, which is upregulated by inflammation, excessive use of alcohol, metabolic syndrome, and tissue damage or turnover (e.g., hepatic or malignancy) (Cullis et al., 2018). Previous studies suggest an association with cognitive decline and (biomarkers of) inflammation (Yaffe et al., 2003; Schram et al., 2007; Sartori et al., 2012). Similar associations are seen with direct or indirect effects of alcohol use, metabolic syndrome, tissue damage, -turnover, or a combination (Brust, 2010; Yates et al., 2012; Janelsins et al., 2014; Nardelli et al., 2019). Notably, the association between serum ferritin and VAT remained independent of adjustment for alcohol use, BMI, hemoglobin, and hs-CRP. Although we tried to fully adjust for these potential confounders, we cannot exclude that these mechanisms, at least in part, might have contributed to the identified association between higher ferritin and lower performance on the VAT score. In the patient setting of neurodegenerative diseases, strong associations of cerebrospinal fluid ferritin have been identified with worse cognitive function in patients with Alzheimer’s disease, patients with Parkinson’s disease, and patients with dementia with Lewy bodies (Ayton et al., 2022). In fact, cerebrospinal fluid ferritin levels even predicted outcomes in patients with Alzheimer’s disease (Ayton et al., 2015, 2017), and could be used as a readout for the inflammatory response during the neurodegenerative phase of Alzheimer’s disease (Brosseron et al., 2021).

Our study has several strengths and limitations. We used a well-phenotyped large cohort of community-dwelling individuals, reflecting a large proportion of the general Dutch population. Moreover, we tried to account as fully as possible for confounders as cognitive functioning is known to be influenced by multiple factors. Limitations of the current study are that cognitive functioning was measured only with two tests, which cover a diverse set of cognitive capabilities but do not reflect performance on all cognitive domains. Although the RFFT is a more sensitive and reliable test for detecting subtle changes in cognitive functioning in both young and old people when compared to tests like the Mini Mental State Examination (MMSE), Trail-Making Test (TMT) or Modified Telephone Interview for Cognitive Status (TICS-M) (Foster et al., 2005; Izaks et al., 2011; Joosten et al., 2014), we are not able to extend our findings to cognitive functioning as a whole. Other tests, e.g., the Rey Auditory Verbal Learning Test (RAVLT) and the Massachusetts General Hospital Cognitive and Physical Functioning Questionnaire (CPFQ) would have given important additional information on cognitive functioning. Another limitation is that we did not have data available on a broader range of iron status parameters, such as hepcidin and soluble transferrin receptor. Finally, a limitation of our study is that we did not identify with biomarkers participants with underlying Alzheimer’s disease and that we did not have availability of a direct measurement of EPO or ferritin in the brain.

In conclusion, this study demonstrates a relatively strong association between higher endogenous EPO levels and better performance on several executive cognitive abilities, as reflected by the RFFT, in the general population. Furthermore, we found that ferritin levels, but not other iron status parameters, were inversely associated with a high performance on VAT scores, reflecting associative memory. Future research should focus on a more comprehensive examination of cognitive functioning, time-dependent relationships, underlying mechanisms, use of brain imaging, identification of patients with Alzheimer’s disease, and opportunities and obstacles for therapeutic interventions.

## Data Availability Statement

The data analyzed in this study is subject to the following licenses/restrictions: Public sharing of individual participant data was not included in the informed consent form of the study, but data can be made available to interested researchers upon reasonable request. Requests to access these datasets should be directed to the data manager of the PREVEND study, Dr. Lyanne Kieneker, l.m.kieneker@umcg.nl.

## Ethics Statement

The study involving human participants was reviewed and approved by Institutional Medical Ethical Review Board of the University Medical Center Groningen. The participants provided their written informed consent to participate in this study.

## Author Contributions

ME realized the research idea and study design and provided supervision and mentorship. GA, MB, SB, and ME carried out data acquisition. GA, MB, and ME carried out statistical analysis. All authors performed the data analysis/interpretation, contributed to important intellectual content during manuscript drafting or revision, and agreed to be personally accountable for the individual’s own contributions and to ensured that questions pertaining to the accuracy or integrity of any portion of the work, even on in which the author was not directly involved, were appropriately investigated and resolved, including documentation in the literature if appropriate.

## Conflict of Interest

JV received consultancy fees from Vifor Pharma. MD has received consultancy fees from Astellas, Kyowa Kirin, Pharmacosmos, Sanofi Genzyme, and Vifor Pharma (all to employer), grant support from Sanofi Genzyme and Vifor Pharma. ME received speakers’ and consultancy fees from Vifor Pharma and served the Advisory Board of Cablon Medical. The remaining authors declare that the research was conducted in the absence of any commercial or financial relationships that could be construed as a potential conflict of interest.

## Publisher’s Note

All claims expressed in this article are solely those of the authors and do not necessarily represent those of their affiliated organizations, or those of the publisher, the editors and the reviewers. Any product that may be evaluated in this article, or claim that may be made by its manufacturer, is not guaranteed or endorsed by the publisher.
